# Nonparametric regression estimator of multivariable Fourier Series for categorical data

**DOI:** 10.1016/j.mex.2024.102983

**Published:** 2024-10-05

**Authors:** Muhammad Zulfadhli, I Nyoman Budiantara, Vita Ratnasari

**Affiliations:** Department of Statistics*, Faculty of Science and Data Analytics,* Institut Teknologi Sepuluh Nopember*, Kampus* ITS-Sukolilo*,* Surabaya 60111*,* Indonesia

**Keywords:** Categorical data, Fourier Series, Nonparametric regression, Maximum Likelihood Estimation

## Abstract

In recent years, Fourier Series estimators in nonparametric regression for quantitative data have received significant attention. However, in reality, there is often a relationship between response and predictor, where the response is categorical data. Some methods developed today to address the case of qualitative response data use only certain approaches. No Fourier Series estimator can handle categorical response data. This paper introduces a new method that uses response variable in the form of categorical data. This study aimed to develop a multivariable Fourier Series nonparametric regression estimator for categorical data. The research methods used are literature and theoretical studies. To apply this method, we used two application data. The results obtained indicate that the nonparametric regression of the Fourier Series provides significantly better estimation results and accuracy for both data applications, due to the small deviance value and larger AUC and Press'Q values. The highlights of this research are summarized below.

• The Fourier Series method for categorical data assumes a relationship between the logit function and predictor variables that has a repeating pattern.
• The estimator was obtained through Maximum Likelihood Estimation and Newton–Raphson method.
• The Fourier Series nonparametric regression method provides better estimation than binary logistic regression.

## Graphical abstract

**Figure:**
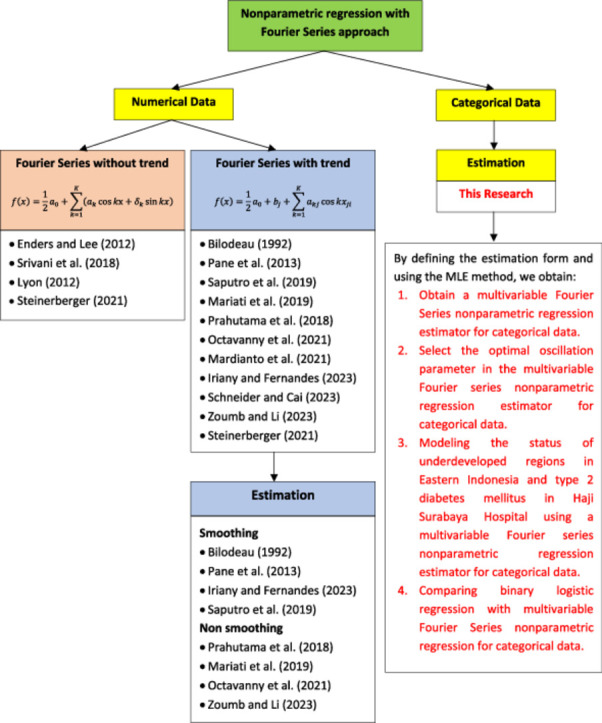

Specifications table

**Table:** 

Subject area:	Mathematics and Statistics
More specific subject area:	Statistics; Nonparametric Regression; Categorical Data
Name of your method:	Maximum Likelihood Estimation
Name and reference of original method:	Fourier Series function developed by Bilodeau (1992), M. Bilodeau, Fourier smoother and additive models, The Canadian Journal of Statistics 20 (1992) 257–269. https://doi.org/10.2307/3315313 Maximum likelihood estimation in the book of Hosmer, D. W., & Lemeshow, S. (2000), Applied Logistic Regression, 2nd Edition. United States of America, Canada. https://books.google.co.id/books?id=Po0RLQ7USIMC&lpg=PP1&pg=PP1#v=onepage&q&f=false
Resource availability:	Respon and predictor variables data of 232 districities/municipalities in East Indonesia can be accessed at the Indonesia publication data website (https://www.bps.go.id/id).

## Background

Nonparametric regression can be used to determine the relationship between the response and predictor variables when the function of the regression curve is unknown. The nonparametric regression curve is assumed to be smooth in the sense that it is contained in a certain function space. The data were expected to find their own form of estimation, without being influenced by the subjective factors of the researcher. Thus, the nonparametric regression approach is highly flexible. The nonparametric approach can be implemented based on observed data using smoothing techniques. There are many smoothing techniques, including the Spline estimator [1], Fourier Series estimator [2], Wavelet estimator [3], Kernel estimator [4], and Local Polynomial [5].

Spline estimators are used for data with changing patterns that depend on knot points [1]. A local polynomial estimator that has been used to reduce the bias properties and asymptotic variance of the local polynomial estimator in nonparametric regression with more than one response variable [6]. Wavelet estimator that has been used to model observations of a signal contaminated with noise, which is Gaussian distributed and additive [7]. The Fourier Series estimator is used for patterned data that tend to repeat [2]. Among these estimators, the Fourier Series method is often used by researchers. This method is very specialized and well used in data cases in which the response and predictor variables exhibit a repeating pattern following a certain trend [8]. The Fourier Series estimator best optimizes the accuracy and computational cost of additive nonparametric regression models [9]. Not only predictors with one predictor variable (univariable) but also predictors with many predictor variables (multivariable) [2,7,10,11].

Fourier Series was first introduced by [2], and then [7] studied the Fourier Series estimator in nonparametric regression. Furthermore [12], applied the Fourier Series in semiparametric regression [13]. developed a birresponse semiparametric regression using Fourier Series, until developed became a Fourier Series nonparametric regression mixture estimator by [[14], [15], [16]] and a Fourier Series semiparametric mixture estimator by [17]. However, previous studies that developed using this method only used quantitative data, such as [[18], [19], [20], [21]]. However, in reality, there is often a relationship between response and predictor, where the response is categorical data. As a result, the Fourier Series nonparametric regression model (quantitative data) developed by the researchers cannot be used to solve the problems associated with qualitative responses.

**Fig. 1:**
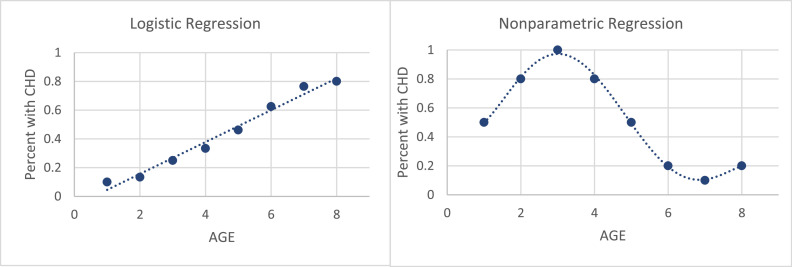
Percentage of subjects with CHD in each group.

Some researchers have developed nonparametric regression estimators for categorical data, such as [22] using Local Likelihood Logit Estimation [23], using the Decision Tree approach, and [24] using the B-Spline function. However, these studies only used certain functions. No previous study has developed a nonparametric regression estimator for categorical response data using a Fourier Series function. A comparison between binary logistic regression and nonparametric regression using categorical data is presented in Fig. 1. An example of a reference we used is the age and coronary heart disease (CHD) status of 100 subjects.

Based on Fig. 1, it is not always the case that the plot follows a linear trend, which sometimes results in non-patterned and repeating patterns. As a result, a conventional nonparametric regression estimator (quantitative data) is not appropriate and tends to produce large errors. In this study, a multivariable Fourier Series nonparametric regression estimator was developed with response variables in the form of categorical data.

## Method details

In obtaining a multivariable Fourier Series nonparametric regression estimator for categorical data, several steps are required: building a Fourier Series nonparametric regression model based on optimal oscillation parameters, then creating a Log Likelihood function and deriving it for each model parameter. Finally, numerical iterations were performed using the Newton–Raphson iteration.

### Probability Distribution

Given $x_{1i},\;x_{2i},\;\ldots \;,\;x_{pi}$ ;i=1,2,...,n, are as many as p predictor variables. Furthermore, the variable $Y_{i}$ is a random Bernoulli distribution variable with a probability distribution of

$$Y_{i}\;∼\;B\left(1,\pi \left(x_{i}\right)\right),\;\pi \left(x_{i}\right)=\pi \left(x_{1i},\;x_{2i},\;\ldots \;,\;x_{pi}\right),\;i=1,2,\ldots ,n$$

where the success probability

$$P\left(Y_{i}\;=\;1\right)=\pi \left(x_{i}\right)$$

and the unsuccessful probability

$$P\left(Y_{i}\;=\;0\right)=1-\pi \left(x_{i}\right)$$

$\pi (x_{i})$ is defined in the probability distribution function $P(Y_{i}\;=\;y_{i})$ as follows.

(1) $$P\left(Y_{i}\;=\;y_{i}\right)=\pi {\left(x_{i}\right)}^{y_{i}}{\left(1-\pi \left(x_{i}\right)\right)}^{1-y_{i}}={\left(\frac{\pi \left(x_{i}\right)}{1-\pi \left(x_{i}\right)}\right)}^{y_{i}}\left(1-\pi \left(x_{i}\right)\right)$$

### Logit function (Link function)

Then, the Eq. (1) can be expressed as a natural logarithmic function (ln)

(2) $$\ln P\left(Y_{i}\;=\;y_{i}\right)=\;y_{i}\;\ln{\left(\frac{\pi \left(x_{i}\right)}{1-\pi \left(x_{i}\right)}\right)}^{\;}+\ln\left(1-\pi \left(x_{i}\right)\right)$$

When made in exponential form, ln function (2) forms an exponential family distribution function as follows.

(3) $$\exp\left(\ln P\left(Y_{i}\;=\;y_{i}\right)\right)\;=\exp\left(y_{i}\;\ln{\left(\frac{\pi \left(x_{i}\right)}{1-\pi \left(x_{i}\right)}\right)}^{\;}+\ln\left(1-\pi \left(x_{i}\right)\right)\right)$$

where, Eq. (3) the exponential family distribution function is defined as follows.

(4) $$f\left(y_{i},\theta \right)=\exp\left(\frac{y_{i}\;\theta -b{\left(\theta \right)}^{\;}}{a\left(\emptyset \right)}+c\left(\theta ,\emptyset \right)\right)$$

Therefore, its probability distribution function belongs to the exponential family of distribution functions.

(5) $$P\left(Y_{i}\;=\;y_{i}\right)=\exp\left(\frac{y_{i}\;\ln{\left(\frac{\pi \left(x_{i}\right)}{1-\pi \left(x_{i}\right)}\right)}^{\;}}{1}+\ln\left(1-\pi \left(x_{i}\right)\right)\right)$$

where,

$$\begin{array}{ccc} \theta & = & \ln\left(\frac{\pi \left(x_{i}\right)}{1-\pi \left(x_{i}\right)}\right)\;a\left(\emptyset \right)\;=1\\ b{\left(\theta \right)}^{\;} & = & \ln\left(1-\pi \left(x_{i}\right)\right)\;c\left(\theta ,\emptyset \right)=0 \end{array}$$

θ in function (5) is a logit function, the logit function for the regression obtained is

(6) $$\theta =\ln\left(\frac{\pi \left(x_{i}\right)}{1-\pi \left(x_{i}\right)}\right)$$

The logit function (link function) simplifies a long regression model and facilitates parameter estimation. To achieve this goal, logit transformation is performed.

### Logit transformation model

The logit transformation model is defined as follows.

(7) $$\ln\left(\frac{\pi \left(x_{i}\right)}{1-\pi \left(x_{i}\right)}\right)=f\left(x_{1i},\;\ldots \;,\;x_{pi}\right)$$

where f is a regression equation or regression function (regression curve) that follows an additive model. Since

$f(x_{1i},\;\ldots \;,\;x_{pi})$ in Eq. (7) can be approximated by a multivariable Fourier Series function as follows.

(8) $$\ln\left(\frac{\pi \left(x_{i}\right)}{1-\pi \left(x_{i}\right)}\right)=\sum_{j=1}^{p}\left(b_{j}x_{ji}+\frac{1}{2}a_{0j}+\sum_{k=1}^{K}a_{kj}\cos kx_{ji}\right);\;i=1,2,\ldots n$$

By using (8), a multivariable Fourier Series nonparametric regression model for categorical data is obtained as follows.

(9) $$\pi \left(x_{i}\right)=\;\frac{e^{\sum_{j=1}^{p}\left(b_{j}x_{ji}+\frac{1}{2}a_{0j}+\sum_{k=1}^{K}a_{kj}\cos kx_{ji}\right)}}{1+e^{\sum_{j=1}^{p}\left(b_{j}x_{ji}+\frac{1}{2}a_{0j}+\sum_{k=1}^{K}a_{kj}\cos kx_{ji}\right)}};\;i=1,2,...,n$$

where, $b_{j},a_{0j}\;\text{and}\;a_{kj}$, j=1,2,…,p, k=1,2,…,K are the model parameters of the Fourier Series function.

### Likelihood function l(β)

The form of the likelihood function

where,

$$\beta =\left(\begin{array}{ccccccccccccc} b_{1} & a_{01} & a_{11} & \ldots & a_{K1} & \vdots & \cdots & \vdots & b_{p} & a_{0p} & a_{1p} & \ldots & a_{\text{Kp}} \end{array}\right)$$

is obtained l(β) using the Maximum Likelihood Estimation (MLE) method.

(10) $$l\left(\beta \right)=\;\prod_{i=1}^{n}P\left(Y_{i}=\;y_{i}\right)=\pi {\left(x_{i}\right)}^{\sum_{i=1}^{n}y_{i}}{\left(1-\pi \left(x_{i}\right)\right)}^{n-\sum_{i=1}^{n}y_{i}}$$

Parameter estimation in logistic regression can be performed using the MLE method by maximizing the first derivative of the log likelihood function. The likelihood function (10) can be easily maximized as follows lnl(β).

### Log-Likelihood function L(β)

(11) $$\begin{array}{ccc} \text{ln}\;\left[l\left(\beta \right)\right] & = & L\left(\beta \right)=\sum_{i=1}^{n}y_{i}\ln\left[\pi {\left(x_{i}\right)}^{\;}\right]+\sum_{i=1}^{n}\left(1-y_{i}\right)\ln\left[1-\pi {\left(x_{i}\right)}^{\;}\right]\\ & = & \;\sum_{i=1}^{n}\left\{y_{i}\left(f\left(x_{1i},\;\ldots \;,\;x_{pi}\right)\right)-\ln\left[1+\exp(f\left(x_{1i},\;\ldots \;,\;x_{pi}\right))\right]\right\} \end{array}$$

The estimator $\hat{\beta }$ is obtained by partially deriving function (11) relative to $b_{j},a_{0j},a_{kj}$ and then equating to 0

$$\frac{\partial L\left(\beta \right)}{\partial b_{j}}=0;\;j=1,2,\ldots ,p$$

$$\frac{\partial L\left(\beta \right)}{\partial a_{0j}}=0;\;j=1,2,\ldots ,p$$

$$\frac{\partial L\left(\beta \right)}{\partial a_{kj}}=0;\;k=1,2,\ldots ,K;\;j=1,2,\ldots ,p$$

The estimator $\hat{b}$ will be obtained using Eq. (12).

(12) $$\sum_{i=1}^{n}\left\{\left(y_{i}-\;\pi \left(x_{i}\right)\right)x_{ji}\right\}=0$$

The estimator $\hat{a_{0}}$ will be obtained using Eq. (13).

(13) $$\sum_{i=1}^{n}\left\{\frac{1}{2}\left(y_{i}-\;\pi \left(x_{i}\right)\right)\right\}=0$$

The estimator $\hat{a_{k}}$ will be obtained using Eq. (14).

(14) $$\sum_{i=1}^{n}\left\{\sum_{k=1}^{K}\cos kx_{ji}\left(y_{i}-\;\pi \left(x_{i}\right)\right)\right\}=0$$

### Newton-raphson iteration

The derivative of L(β) (11) against $b_{j},a_{0j},a_{kj}$ that has been made in the implicit equation, gives results that are not closed form, so it is necessary to continue with the numerical iteration method using the Newton–Raphson method.

(15) $$\beta^{\left(t+1\right)}=\;\beta^{\left(t\right)}-\;{\left(H{\left(\beta \right)}^{\left(t\right)}\right)}^{-1}g{\left(\beta \right)}^{\left(t\right)}$$

where $\beta^{\left(t\right)}$ is the β of the *t*-th iteration*, t* *=* *1,2…,*convergent.

$$\beta^{\left(t\right)}=\left(\begin{array}{ccccccccccccc} b_{1}^{\left(t\right)} & a_{01}^{\left(t\right)} & a_{11}^{\left(t\right)} & \ldots & a_{K1}^{\left(t\right)} & \vdots & \cdots & \vdots & b_{p}^{\left(t\right)} & a_{0p}^{\left(t\right)} & a_{1p}^{\left(t\right)} & \ldots & a_{Kp}^{\left(t\right)} \end{array}\right)$$

g(β) is the gradient vector of β

(16) $$g\left(\beta \right)=\;{\left(\frac{\partial L\left(\beta \right)}{\partial b_{1}},\frac{\partial L\left(\beta \right)}{\partial a_{01}},\frac{\partial L\left(\beta \right)}{\partial a_{11}},\cdots ,\frac{\partial L\left(\beta \right)}{\partial a_{K1}},\cdots ,\frac{\partial L\left(\beta \right)}{\partial b_{p}},\frac{\partial L\left(\beta \right)}{\partial a_{0p}},\frac{\partial L\left(\beta \right)}{\partial a_{1p}},\cdots ,\frac{\partial L\left(\beta \right)}{\partial a_{Kp}}\right)}^{T}$$

and H(β) is the Hessian matrix of β in function (16), with the following equation.

(17) $$H\left(\beta \right)\;=\left[\begin{array}{cc} \begin{array}{cc} \frac{\partial^{2}L\left(\beta \right)}{\partial {b_{1}}^{2}} & \frac{\partial^{2}L\left(\beta \right)}{\partial b_{1}\partial a_{01}}\\ \frac{\partial^{2}L\left(\beta \right)}{\partial a_{01}\partial b_{1}} & \frac{\partial^{2}L\left(\beta \right)}{\partial {a_{01}}^{2}} \end{array} & \begin{array}{cc} \cdots & \frac{\partial^{2}L\left(\beta \right)}{\partial b_{1}\partial a_{Kp}}\\ \cdots & \frac{\partial^{2}L\left(\beta \right)}{\partial a_{01}\partial a_{Kp}} \end{array}\\ \begin{array}{cc} \vdots & \vdots \\ \frac{\partial^{2}L\left(\beta \right)}{\partial a_{Kp}\partial b_{1}} & \frac{\partial^{2}L\left(\beta \right)}{\partial a_{Kp}\partial a_{01}} \end{array} & \begin{array}{cc} \ddots & \vdots \\ \cdots & \frac{\partial^{2}L\left(\beta \right)}{\partial {a_{Kp}}^{2}} \end{array} \end{array}\right]$$

The elements of vector g(β) (16) are obtained from the first derivative of function L(β) with respect to $b_{j},a_{0j},a_{kj}$, while elements of matrix H(β) (17) are obtained from the second derivative of function L(β) with respect to $b_{u},a_{0u},a_{ku}$.

*Second Derivative of* L(β) *Function with Respect to* $b_{u}$

(18) $$\frac{\partial^{2}L\left(\beta \right)}{\partial b_{u}\partial b_{j}}=\;-\sum_{i=1}^{n}x_{ji}x_{ui}\pi \left(x_{i}\right)\left(1-\pi \left(x_{i}\right)\right)$$

In the same manner (18), the second derivative of the parameter combination is obtained as follows.

(19) $$\frac{\partial^{2}L\left(\beta \right)}{\partial a_{ku}\partial b_{j}}=\;-\sum_{i=1}^{n}\sum_{k=1}^{K}\pi \left(x_{i}\right)\left(1-\pi \left(x_{i}\right)\right)x_{ji}\cos kx_{ui}$$

*Second Derivative of* L(β) *Function with Respect to* $a_{0u}$

(20) $$\frac{\partial^{2}L\left(\beta \right)}{\partial a_{0u}\partial a_{0j}}=\;-\frac{1}{4}\sum_{i=1}^{n}\pi \left(x_{i}\right)\;\left(1-\pi \left(x_{i}\right)\right)$$

In the same manner (20), the second derivative of the parameter combination is obtained as follows.

(21) $$\frac{\partial^{2}L\left(\beta \right)}{\partial a_{ku}\partial a_{0j}}=\;-\frac{1}{2}\sum_{i=1}^{n}\sum_{k=1}^{K}\pi \left(x_{i}\right)\left(1-\pi \left(x_{i}\right)\right)\cos kx_{ui}$$

*Second Derivative of* L(β) *Function with Respect to* $a_{ku}$

(22) $$\frac{\partial^{2}L\left(\beta \right)}{\partial a_{ku}\partial a_{kj}}=\;-\sum_{i=1}^{n}\sum_{k=1}^{K}\cos kx_{ji}\sum_{k=1}^{K}\cos kx_{ui}\pi \left(x_{i}\right)\left(1-\pi \left(x_{i}\right)\right)$$

In the same manner (22), the second derivative of the parameter combination is obtained as follows.

(23) $$\frac{\partial^{2}L\left(\beta \right)}{\partial a_{0u}\partial a_{kj}}=-\frac{1}{2}\sum_{i=1}^{n}\sum_{k=1}^{K}\pi \left(x_{i}\right)\left(1-\pi \left(x_{i}\right)\right)\cos kx_{ji}$$

where, $b_{j},a_{0j}\;\text{and}\;a_{kj}$, j,u=1,2,…,p, j≠u, k=1,2,…,K are the model parameters of the Fourier Series function.

### Estimator $\hat{\beta }$

From the Newton-Raphson iteration equation, $\hat{\beta }$ will be obtained when

(24) $$\left|\beta^{\left(t+1\right)}-\beta^{\left(t\right)}\right|<\varepsilon ,\;\varepsilon =0,000001$$

Thus, the estimator $\hat{\beta }$ is given by

$$\hat{\beta }=\left(\begin{array}{ccccccccccccc} {\hat{b}}_{1} & {\hat{a_{0}}}_{1} & {\hat{a_{1}}}_{1} & \ldots & {\hat{a_{K}}}_{1} & \vdots & \cdots & \vdots & {\hat{b}}_{p} & {\hat{a_{0}}}_{p} & {\hat{a_{1}}}_{p} & \ldots & {\hat{a_{K}}}_{p} \end{array}\right)$$

Based on the result of the estimator $\hat{\beta }$, multivariable Fourier Series nonparametric regression model for categorical data can be written:

(25) $$\hat{\pi }\left(x_{i}\right)=\;\frac{e^{{\hat{b}}_{1}x_{1i}+\frac{1}{2}{\hat{a}}_{01}+{\hat{a}}_{11}\cos x_{1i}+\ldots +{\hat{a}}_{K1}\cos Kx_{1i}+\ldots +{\hat{b}}_{p}x_{pi}+\frac{1}{2}{\hat{a}}_{0p}+{\hat{a}}_{1p}\cos x_{pi}+\ldots +{\hat{a}}_{Kp}\cos Kx_{pi}}}{1+e^{{\hat{b}}_{1}x_{1i}+\frac{1}{2}{\hat{a}}_{01}+{\hat{a}}_{11}\cos x_{1i}+\ldots +{\hat{a}}_{K1}\cos Kx_{1i}+\ldots +{\hat{b}}_{p}x_{pi}+\frac{1}{2}{\hat{a}}_{0p}+{\hat{a}}_{1p}\cos x_{pi}+\ldots +{\hat{a}}_{Kp}\cos Kx_{pi}}}$$

where, ${\hat{b}}_{1},{\hat{a}}_{01}\;\text{and}\;{\hat{a}}_{k1}\;$are the estimator model of the Fourier Series function for predictor variable $x_{1}$, while ${\hat{b}}_{p},{\hat{a}}_{0p}\;\text{and}\;{\hat{a}}_{kp}\;$are the estimator model of the Fourier Series function for predictor variable $x_{p}$, where K is the number of oscillation parameters and p is the number of predictor variables.

## Method validation

In applying the multivariable Fourier Series nonparametric regression method for categorical data, we used 2 application data: the status of underdeveloped regions in Eastern Indonesia in 2021 and the status of diabetes at the internal medicine department of Hajj Hospital, Surabaya, 2018.

### The status of underdeveloped regions in Eastern indonesia in 2021

We used secondary data, which consists of 1 response variable (y) and 6 predictor variables (x). Underdeveloped region is the current status of districts whose regions and communities are less developed than other regions on a national scale (Ministerial Regulation No. 11/2020). All data were collected from the Ministry of Villages, Development of Underdeveloped Regions, and Transmigration, Ministry of Home Affairs, Ministry of Education, Culture, Research and Technology, Ministry of Finance, and the Central Bureau of Statistics of the Republic of Indonesia. The variables are detailed in Table 1.

**Table 1:** Variable description.

Variable	Notation	Description	Unit	Scale
Response	y	Status of Underdeveloped Regions	0 = Developed 1 = Underdeveloped	Nominal
Predictor	$x_{1}$	Percentage of Households Using Clean Water	Percent	Ratio
	$x_{2}$	Percentage of Villages with the Widest Main Road Surface Type Asphalt/Concrete	Percent	Ratio
	$x_{3}$	Percentage of Villages without Disaster	Percent	Ratio
	$x_{4}$	GDRB per Capita	Thousand rupiahs	Ratio
	$x_{5}$	Percentage Senior High School Enrollment Rate	Percent	Ratio
	$x_{6}$	PAD per Capita	Hundred thousand rupiahs	Ratio

**Fig. 2:**
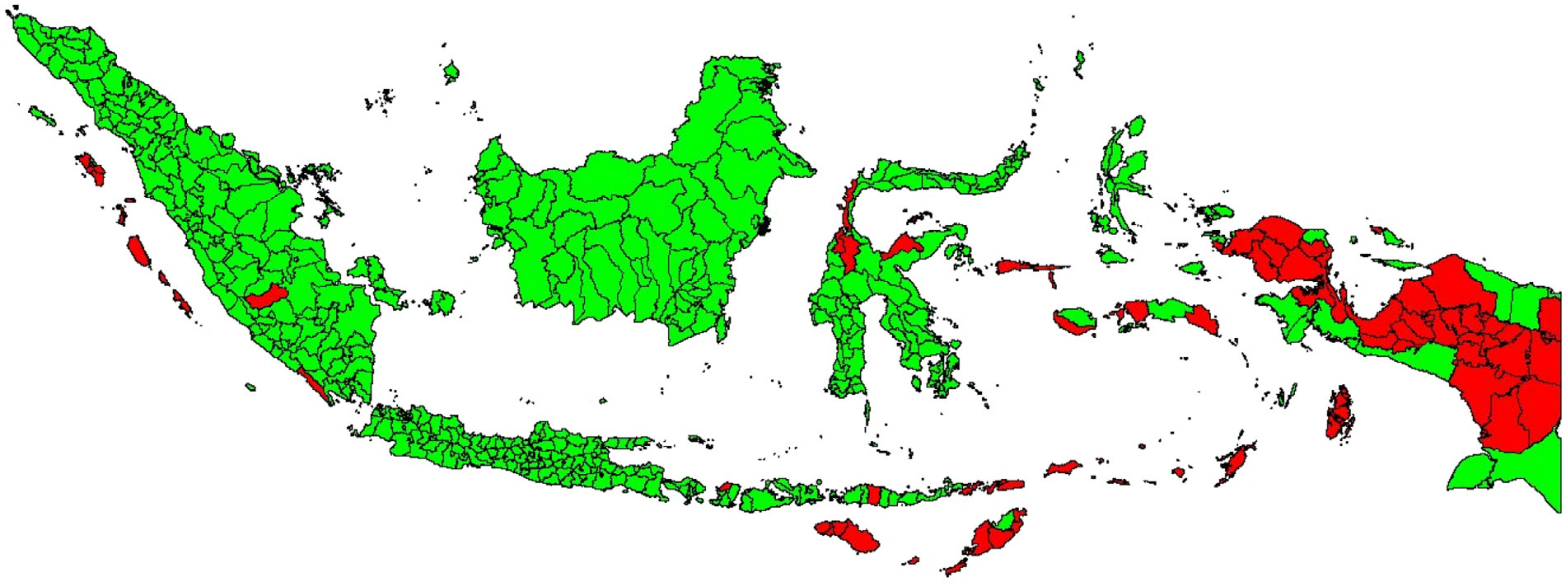
Distribution of underdeveloped regions in indonesia in 2021.

A map of the distribution of underdeveloped regions is presented in Fig. 2 below:

Based on Fig. 1, 62 underdeveloped regencies exist in Indonesia, 55 of which are in Eastern Indonesia, as shown in Table 2 below.

**Table 2:** List of underdeveloped regions in Eastern indonesia 2021 according to presidential regulation no. 63/2020.

Region	Province	District	Total
Papua	Papua Barat	Teluk Wondama, Teluk Bintuni, Sorong Selatan, Sorong, Tambrauw, Maybrat, Manokwari Selatan, Pegunungan Arfak	8 Districts
	Papua	Jayawijaya, Nabire, Paniai, Puncak Jaya, Boven Digoel, Mappi, Asmat, Yahukimo, Pegunungan Bintang, Tolikara, Keerom, Waropen, Supiori, Memberamo Raya, Nduga, Lanny Jaya, Memberamo Tengah, Yalimo, Puncak, Dogiyai, Intan Jaya, dan Deiyai	22 Districts
Maluku	Maluku	Maluku Tenggara Barat, Kepulauan Aru, Seram Bagian Barat, Seram Bagian Timur, Maluku Barat Daya, Buru Selatan	6 Districts
	Maluku Utara	Kepulauan Sula, Pulau Taliabu.	2 Districts
Nusa Tenggara	NTB	Lombok Utara	1 District
	NTT	Sumba Barat, Sumba Timur, Kupang, Timor Tengah Selatan, Belu, Alor, Lembata, Rote Ndao, Sumba Tengah, Sumba Barat Daya, Manggarai Timur, Sabu Raijua, Malaka.	13 Districts
Sulawesi	Sulawesi Tengah	Donggala, Tojo Una-Una, Sigi	3 Districts
**Total**	**11 Provinces**	**55 Districts**	

### Descriptive analytics

Descriptive analysis is used to determine the characteristics of the data for each variable as follows Table 3.

**Table 3:** Descriptive statistics of research variables.

Variable	Mean	Variance	Min	Max
y	–	–	–	–
$x_{1}$	81.28	373.37	0.00	100.00
$x_{2}$	69.14	821.01	0.00	100.00
$x_{3}$	55.90	681.78	0.00	100.00
$x_{4}$	55.20	3937.56	6.18	589.11
$x_{5}$	63.89	246.68	8.25	94.92
$x_{6}$	46.93	1191.44	4.81	281.79

Table 3 provides information about the characteristics of the variables, which represents the status of underdeveloped regions in 232 regencies/cities in Indonesia. In addition, each independent variable is correlated with the dependent variable [25], does not have missing values [26], and there is no multicollinearity between independent variables [27]. The explanation for each predictor variable used in this study is as follows (Ministerial Regulation No. 11/2020).

1. Percentage of Households Using Clean Water in Each District/City, is the number of households using clean water divided by the number of households in the district concerned multiplied by one hundred percent (100 %).
2. Percentage of Villages with the Widest Main Road Surface Type of Asphalt/Concrete in Each Regency/City, is the number of villages with asphalt/concrete main road surface type divided by the number of villages in the district multiplied by one hundred percent (100 %).
3. Percentage of Villages without Disaster in Each District/City*,* is the number of villages that have not experienced landslides, floods, flash floods, earthquakes, tsunamis, sea tides, whirlwinds/tornadoes/typhoons, volcanic eruptions, forest/land fires or land droughts in the last 3 (three) years divided by the total number of villages in the relevant district multiplied by one hundred percent (100 %).
4. GRDP Per-Capita in Each Regency/City, is the corrected value of gross regional domestic product in the kabupaten divided by the total population in the district.
5. Percentage Senior High School Enrollment Rate in Each District/City, is the number of people aged 16–18 (sixteen to eighteen) years who are currently attending high school education compared to the total population aged 16–18 (sixteen to eighteen) years multiplied by one hundred percent (100 %).
6. PAD Per-Capita in Each Regency/City, is the value of the district's own-source revenue divided by the total population in the district.

### Binary logistic regression model

The binary logistic regression model is as follows.

(26) $$\pi \left(x_{i}\right)=\;\frac{e^{\beta_{0}+\sum_{l=1}^{p}\beta_{l}x_{li}}}{1+e^{\beta_{0}+\sum_{l=1}^{p}\beta_{l}x_{li}}},\;i=1,2,...,n$$

Where l is the number of predictor variables.

### Parameter estimation

Based on binary logistic regression model (25), the results of parameter estimation in the binary logistic regression model for data on the status of underdeveloped regions in Eastern Indonesia in 2021 are as follows.

$$\hat{\pi }\left(x_{i}\right)=\;\frac{e^{3.6276-0.0084x_{1i}-0.0438x_{2i}+0.0128x_{3i}-0.0121x_{4i}-0.0072x_{5i}\;-0.0301x_{6i}}}{1+e^{3.6276-0.0084x_{1i}-0.0438x_{2i}+0.0128x_{3i}-0.0121x_{4i}-0.0072x_{5i}\;-0.0301x_{6i}}}$$

More details can be seen in Table 4.

**Table 4:** Parameter Estimation in binary logistic regression.

Parameters	Estimations
$\beta_{0}$	3.6276
$\beta_{1}$	−0.0085
$\beta_{2}$	−0.0438
$\beta_{3}$	0.0128
$\beta_{4}$	−0.0121
$\beta_{5}$	−0.0072
$\beta_{6}$	−0.0301

### Fourier Series nonparametric regression model

**Fig. 3:**
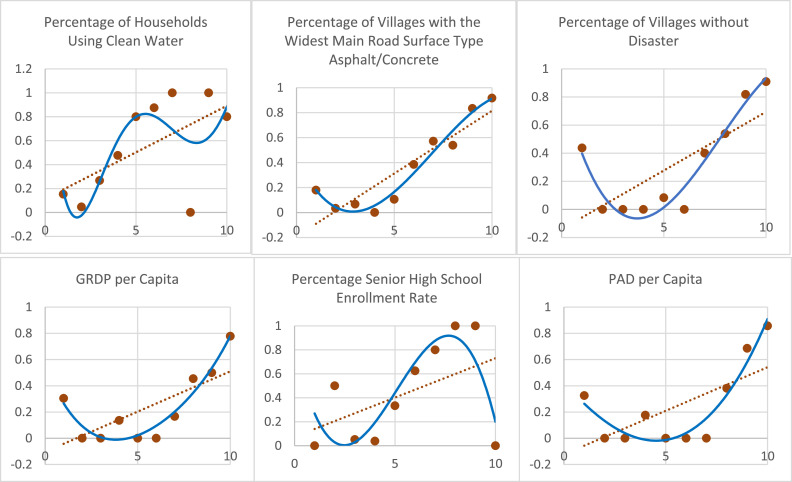
Scatterplots of several data groups versus the number of underdeveloped regions in each group.

We created a scatterplot for each predictor variable that was built into several groups versus the presentation of the number of underdeveloped regions in each group to identify the relationship that followed the Fourier Series nonparametric regression model. The scatterplot is presented in Fig. 3 as follows.

Based on Fig. 3, the probability of underdeveloped regions for each independent variable increases, and a repeating pattern follows an upward trend line.

### Selecting optimal oscillation parameters

The oscillation parameters in the Fourier Series nonparametric regression estimator were selected based on the smallest AIC value. The number of oscillation parameters used in this study was limited to produce a model that is not too complicated and provides appropriate significance results. With the help of the R algorithm, the AIC results for each combination of oscillation parameters in the model are given in Table 5.

**Table 5:** Minimum AIC results for each number of oscillation parameter.

Number of Oscillation Parameter	Oscillation Parameter Combination (K)						AIC (K)
	$x_{1}$	$x_{2}$	$x_{3}$	$x_{4}$	$x_{5}$	$x_{6}$	
*K* = 1	1	1	1	1	1	1	169.3606
*K* = 2	1	2	1	1	1	1	169.1771
*K* = 3	3	2	1	1	1	1	**168.4055**
*K* = 4	3	2	1	1	1	1	168.4055

Based on Table 5, the model with a combination of oscillation parameters 3,2,1,1,1,1 is the model with optimal oscillation parameters because it has the smallest AIC value.

### Parameter estimation

Based on the Fourier Series nonparametric regression model (9), the results of parameter estimation in the multivariable Fourier Series nonparametric regression model for data on the status of underdeveloped regions in Eastern Indonesia in 2021 as follows.

$$\hat{\pi }\left(x_{i}\right)=\;\frac{e^{4.03-0.0005x_{1i}+0.09\cos x_{1i}+0.40\cos2x_{1i}-0.55\cos3x_{1i}+\ldots -0.03x_{6i}+0.08\cos x_{6i}}}{1+e^{4.03-0.0005x_{1i}+0.09\cos x_{1i}+0.40\cos2x_{1i}-0.55\cos3x_{1i}+\ldots -0.03x_{6i}+0.08\cos x_{6i}}}$$

More details can be seen in Table 6.

**Table 6:** Parameter Estimation in Fourier Series nonparametric regression.

Parameters	Estimations	Parameters	Estimations
$a_{0}$	4.03933	$b_{3}$	0.011595
$b_{1}$	0.000514	$a_{1,3}$	−0.45005
$a_{1,1}$	0.094677	$b_{4}$	−0.00925
$a_{2,1}$	0.407124	$a_{1,4}$	−0.34811
$a_{3,1}$	−0.55419	$b_{5}$	−0.01175
$b_{2}$	−0.05826	$a_{1,5}$	0.396991
$a_{1,2}$	−0.54542	$b_{6}$	−0.03384
$a_{2,2}$	−0.51361	$a_{1,6}$	0.083197

### The status of diabetes at the internal medicine of the Hajj Hospital Surabaya 2018

We used secondary data on 60 outpatients with type 2 diabetes mellitus treated at the internal medicine department of Hajj Hospital, Surabaya from August 2018. The data consists of 1 response variable (y) and 3 predictor variables (x). According to the World Health Organization, type 2 diabetes mellitus is characterized by increased blood glucose levels at two examinations accompanied by clinical symptoms. All data were collected from a patient at Surabaya Hajj General Hospital. The variables are detailed in Table 7.

**Table 7:** Variable description.

Variable	Notation	Description	Unit	Scale
Response	y	Incidence of Type 2 Diabetes Mellitus	0 = Non Diabetes 1 = Diabetes	Nominal
Predictor	$x_{1}$	Age	year	Ratio
	$x_{2}$	Body Mass Index	kg/$m_{2}$	Ratio
	$x_{3}$	Waist Circumference	cm	Ratio

### Descriptive analytics

Descriptive analysis is used to determine the characteristics of the data for each variable as shown in Table 8.

**Table 8:** Descriptive Statistics of Research Variables.

Variable	Mean	Variance	Min	Max
y	–	–	–	–
$x_{1}$	57.4	198.75	17.00	83.00
$x_{2}$	24.41	17.15	16.02	33.78
$x_{3}$	90.60	121.84	64.00	119.00

Table 8 provides information about the characteristics of the variables, namely, the status of diabetes at the internal medicine department of Hajj Hospital, Surabaya. In addition, each variable does not have missing values, and no multicollinearity between independent variables. The explanation for each predictor variable used in this study is explained as follows (World Health Organization).

1. Age, according to the new age classification, the young age is from 25 to 44 years, middle age is 44–60, elderly age is 60–75, senile age is 75–90 and long-lived individuals are after 90. This transition poses health risks.
2. Body Mass Index (BMI) is a measure of obesity, and obesity is associated with diabetes mellitus. The major adult BMI classifications were as follows: underweight (under 18.5 kg/m^2^), normal weight (18.5 to 24.9), overweight (25 to 29.9), and obese (30 or more).
3. Waist circumference is one of the anthropometric measurements of abdominal or visceral obesity and is associated with morbidity and mortality due to obesity, such as diabetes mellitus. Waist circumference should be used to refine action levels based on BMI.

### Binary logistic regression model

*Parameter Estimation*

Based on binary logistic regression model (25), the results of parameter estimation in the binary logistic regression model for data on diabetes status at the internal medicine department of Hajj Hospital in Surabaya are as follows.

$$\hat{\pi }\left(x_{i}\right)=\;\frac{e^{-11.7819+0.1181x_{1i}+0.1320x_{2i}+0.0265x_{3i}}}{1+e^{-11.7819+0.1181x_{1i}+0.1320x_{2i}+0.0265x_{3i}}}$$

More details can be seen in Table 9.

**Table 9:** Parameter Estimation in binary logistic regression.

Parameters	Estimations
$\beta_{0}$	−11.7819
$\beta_{1}$	0.1181
$\beta_{2}$	0.1320
$\beta_{3}$	0.0265

### Fourier Series nonparametric regression model

**Fig. 4:**
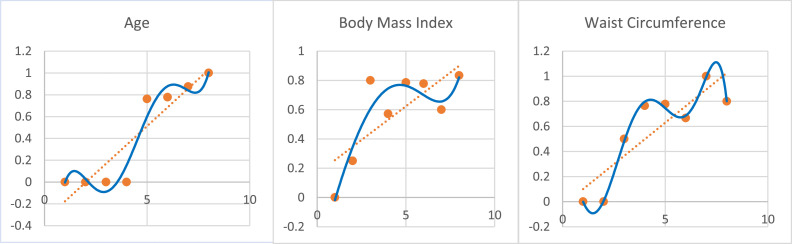
Scatterplots of several groups of data versus the incidence of diabetes in each group.

The scatterplot is presented in Fig. 4 as follows.

Based on Fig. 4, the probability of incidence of diabetes for each independent variable increased, and there was a repeating pattern following the trend line.

*Selecting Optimal Oscillation Parameters*

The AIC results for each combination of oscillation parameters in the model are given in Table 10.

**Table 10:** Minimum AIC results for each number of oscillation parameter.

Number of Oscillation Parameter	Oscillation Parameter Combination (K)			AIC (K)
	$x_{1}$	$x_{2}$	$x_{3}$	
*K* = 1	1	1	1	61.5431
*K* = 2	1	2	1	57.8379
*K* = 3	1	2	1	57.8379
*K* = 4	4	3	4	**54.0027**

Based on Table 10, the model with a combination of oscillation parameters 4,3,4 is the model with optimal oscillation parameters because it has the smallest AIC value.

### Parameter estimation

Based on Fourier Series nonparametric regression model (9), the results of the parameter estimation in the multivariable Fourier Series nonparametric regression model for data on the status of diabetes at the internal medicine of the Hajj Hospital Surabaya as follows.

$$\hat{\pi }\left(x_{i}\right)=\;\frac{e^{-33.45+0.55x_{1i}+4.79\cos x_{1i}-0.87\cos2x_{1i}-2.09\cos3x_{1i}-6.49\cos4x_{1i}+\ldots -0.29x_{3i}-4.55\cos x_{3i}+0.36\cos2x_{3i}-3.51\cos3x_{3i}-6.74\cos4x_{3i}}}{1+e^{-33.45+0.55x_{1i}+4.79\cos x_{1i}-0.87\cos2x_{1i}-2.09\cos3x_{1i}-6.49\cos4x_{1i}+\ldots -0.29x_{3i}-4.55\cos x_{3i}+0.36\cos2x_{3i}-3.51\cos3x_{3i}-6.74\cos4x_{3i}}}$$

More details can be seen in Table 11.

**Table 11:** Parameter estimation in Fourier Series nonparametric regression.

Parameters	Estimations	Parameters	Estimations	Parameters	Estimations
$a_{0}$	−33.4560	$a_{1,4}$	−6.4988	$b_{3}$	−0.2911
$b_{1}$	0.5537	$b_{2}$	1.2839	$a_{3,1}$	−4.5566
$a_{1,1}$	4.7961	$a_{2,1}$	0.5799	$a_{3,2}$	0.3602
$a_{1,2}$	−0.8740	$a_{2,2}$	1.0119	$a_{3,3}$	−3.5174
$a_{1,3}$	−2.0921	$a_{3,3}$	5.4764	$a_{3,4}$	−6.7466

### Comparison of binary logistic regression and Fourier Series nonparametric regression

*Getting the Best Model Based on Deviance Value*

The regression model chosen is the model that has the smallest deviance value. Using the deviance statistical test, the following results are obtained in Table 12.

**Table 12:** Comparison of deviance values.

Methods	Deviance Values	
	Case I	Case 2
Binary Logistic Regression	151.8901	53.0073
Fourier Series Nonparametric Regression	136.4056	24.0027

Based on Table 12, the deviance value for the Fourier Series nonparametric regression (136.4056 & 53.0073) was smaller than that for the binary logistic regression (151.8901 & 24.0027). Therefore, the Fourier Series nonparametric regression model is the best model for data on the status of underdeveloped regions in 2021 and the status of diabetes at the internal medicine department of Hajj Hospital Surabaya in 2018 because it has the smallest deviance value.

*Getting the Best Classification Based on AUC & Press's Q Value*

The selected Fourier Series nonparametric regression model demonstrated the highest AUC or the smallest Press's Q. Using the classification test, the following results are obtained in Table 13.

**Table 13:** Comparison of classification test.

Case	Methods	Accuracy	Sensitivity	Specificity	AUC	Press's Q value	Chi Square
Case 1	Binary Logistic Regression	84.48 %	94.35 %	52.73 %	73.53 %	110.3448	3.8414
	Fourier Series Nonparametric Regression	87.50 %	95.48 %	61.81 %	78.65 %	130.5000	3.8414
Case 2	Binary Logistic Regression	73.33 %	42.86 %	89.74 %	66.30 %	13.0666	3.8414
	Fourier Series Nonparametric Regression	85.00 %	76.19 %	89.74 %	82.97 %	29.4000	3.8414

**Fig. 5:**
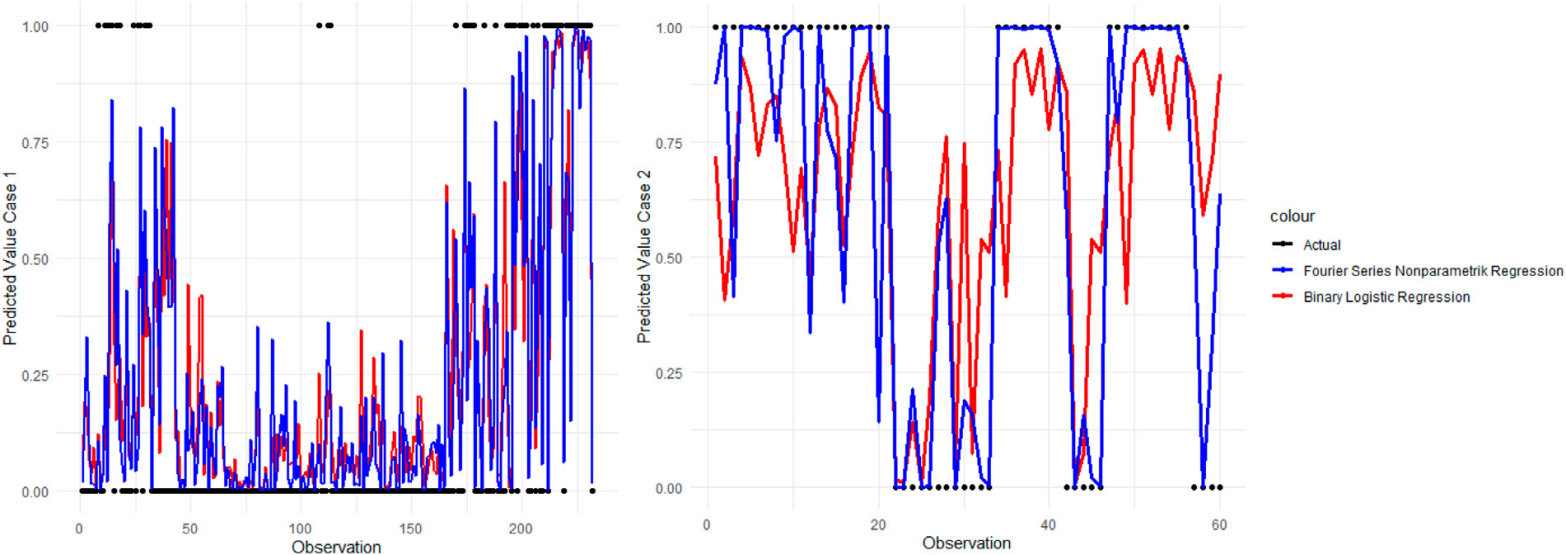
Comparison of estimated value.

Based on Table 13, case 1 shows that the AUC value of the Fourier Series nonparametric regression (78.65 %) is higher than binary logistic regression (73.53 %). In addition, a larger Press's Q value for nonparametric regression (130.5000) indicates that the Fourier Series nonparametric regression model can classify well and has a greater chance of rejecting H0 or Press's Q > Chi Square. As in case 2, the AUC value of Fourier Series nonparametric regression (82.97 %) was greater than binary logistic regression (66.30 %). In addition, a larger Press's Q value for nonparametric regression (29.4000) indicates that the Fourier Series nonparametric regression model can classify well and has a greater chance of rejecting H0 or Press's Q > Chi Square. The comparison using the estimated value plot is shown in Fig. 5.

Based on Fig. 5, the estimated value plots show that the accuracy values of the two methods fluctuated and did not necessarily indicate which method was better. However, for certain cases, such as the one used in this paper, Fourier Series nonparametric regression tends to perform better than binary logistic regression. The binary logistic regression model was superior for specific observations. The comparison through running time is shown in Table 14.

**Table 14:** Comparison of running time.

Case	Methods	The number of oscillation parameters	Running Time
Case 1	Binary Logistic Regression	–	00.50 s
	Fourier Series Nonparametric Regression	*K* = 1	00.48 s
		*K* = 2	05.23 s
		*K* = 3	2 min 10 s
		*K* = 4	1 hour 58 min 11 s
Case 2	Binary Logistic Regression	–	00.44 s
	Fourier Series Nonparametric Regression	*K* = 1	00.38 s
		*K* = 2	00.58 s
		*K* = 3	01.43 s
		*K* = 4	04.21 s

Based on Table 14, Fourier Series nonparametric regression gives longer results than binary logistic regression. Due to the process of selecting the optimal oscillation parameters, Fourier Series nonparametric regression is highly dependent on the number of variables and the number of combinations of oscillation parameters.

## Conclusion

The Fourier Series nonparametric regression built for modeling the relationship between response and predictor, where the response is categorical data and have a repeating pattern. The model can be approximated using the bernoulli distribution and constructed based on the MLE method. The use of the model for modeling the status of underdeveloped regions in Eastern Indonesia and the status of diabetes at the internal medicine department of Hajj Hospital Surabaya. The results show that the Fourier Series nonparametric regression model performs better than the binary logistic regression model.

## Limitations

1. The method used to select the optimal knot point was the smallest AIC.
2. The number of oscillation parameters (k) of the Fourier Series in this study is k = 1, 2, 3, and 4.
3. The estimation method used in the Fourier Series nonparametric regression model of categorical data is Maximum Likelihood Estimation (MLE).
4. The best model comparison method used the classification accuracy test (AUC & Press's Q).
5. This method uses two data sources. The first data were secondary data on the status of underdeveloped regions in Eastern Indonesia in 2021 (West Nusa Tenggara, East Nusa Tenggara, West Kalimantan, Central Kalimantan, South Kalimantan, East Kalimantan, North Kalimantan, North Sulawesi, Central Sulawesi, South Sulawesi, Southeast Sulawesi, Gorontalo, West Sulawesi, Maluku, North Maluku, West Papua, and Papua). The second data were secondary data on the status of diabetes at the internal medicine department of Hajj Hospital Surabaya in 2018.

## Ethics statements

The data used in this research were secondary data collected from the publications of each province in Eastern Indonesia and internal medicine at Hajj Hospital, Surabaya.

## CRediT authorship contribution statement

**Muhammad Zulfadhli:** Conceptualization, Methodology, Software, Writing – original draft, Visualization. **I Nyoman Budiantara:** Conceptualization, Methodology, Writing – review & editing, Validation, Supervision. **Vita Ratnasari:** Conceptualization, Methodology, Writing – review & editing, Validation, Supervision.

## Declaration of competing interest

The authors declare that they have no known competing financial interests or personal relationships that could have appeared to influence the work reported in this paper.

## Data Availability

The authors do not have permission to share data.

## References

[1] Eubank R.L. (1998).

[2] Bilodeau M. (1992). Fourier smoother and additive models. Canadian of Statistic.

[3] Li L. (2002). Nonlinear Waveled-Based Nonparametric Curve Estimation with Consored Data and Inference on Long Memory Processes. ProQuest Information and Learning Company. United States Code.

[4] Okumura H., Naito K. (2006). Non-parametric Kernel Regression for multinomial data. J. Multivar. Anal..

[5] Sua L., Ullah A. (2008). Local polynomial estimation of nonparametric simultaneous equations models. J. Econom..

[6] Welsh A.H., Yee T.W. (2006). Local regression for vector responses. J. Stat. Plan. Inference.

[7] Antoniadis A., Bigot J., Spatinas T. (2001). Wavelet estimators in nonparametric regression: a comparative simulation study. J. Stat. Softw..

[8] Tripena A., Budiantara I.N. (2007). Fourier estimator in nonparametric regression. Int. Conference On Natural Sci. Appl. Natural Sci..

[9] Amato U., Antoniadis A., De Feis I. (2002). Fourier series approximation of separable models. J. Comput. Appl. Math..

[10] Silverberg J., Morton J. (2007). Fourier series of half-range functions by smooth extension. J. Appl. Math. Modell..

[11] De Canditiis D., De Feis I. (2004). Pointwise convergence of fourier regularization for smoothing data. J. Comput. Appl. Math..

[12] Asrini L.J., Budiantara I.N. (2014). Fourier Series semiparametric regression models (Case Study: the Production of Lowland Rice Irrigation in Central Java). ARPN J. Eng. Appl. Sci..

[13] Budiantara I.N., Ratnasari V., Zain I., Ratna M., Mardianto M.F.F. (2015). Modeling of HDI and PQLI in East Java (Indonesia) using biresponse semiparametric regression with fourier series approach. Asian Transact. Basic Appl. Sci. J..

[14] Sudiarsa I.W., Budiantara I.N., Purnami S.W. (2015). Combined estimator fourier series and spline truncated in multivariable nonparametric regression. Appl. Math. Sci..

[15] Ratnasari V., Budiantara I.N., Zain I., Ratna M., Mariati N.P.A.M. (2015). Comparison truncated spline and fourier series in multivariable nonparametric regression models (Application: data of Poverty in Papua, Indonesia). Int. J. Basic and Appl. Sci..

[16] Budiantara I.N., Ratnasari V., Ratna M., Wibowo W., Afifah N., Rahmawati D.P., Octavanny M.A.D (2019). Modeling percentage of poor people in indonesia using kernel and fourier series mixed estimator in nonparametric regression. Invest. Operacional.

[17] Nisa K., Budiantara I.N., Rumiati A.T. (2017). IOP Conference Series: Earth and Environmental Science.

[18] Ampa A.T., Budiantara I.N., Zain I. (2022). Modeling the level of drinking water clarity in surabaya city drinking water regional company using combined estimation of multivariable Fourier Series and Kernel. Sustainability.

[19] Ramli M., Budiantara I.N., Ratnasari V. (2023). A method for parameter hypothesis testing in nonparametric regression with Fourier Series Approach. MethodsX.

[20] Jou P.H., Mirhashemi S.H. (2023). Frequency analysis of extreme daily rainfall over an arid zone of iran using Fourier Series method. Appl. Water Sci..

[21] Laome L., Budiantara I.N., Ratnasari V. (2024). AIP Conference Proceedings.

[22] Suliyanto S., Rifada M., Tjahjono E. (2020). Symposium on Biomathematics 2019.

[23] Hamie H., Hoayek A., El-Ghoul B., Khalifeh M. (2022). Application of non-parametric statistical methods to predict pumpability of geopolymers for well cementing. J. Pet. Sci. Eng..

[24] Wang T., Tang W., Lin Y., Su W. (2023). Semi-supervised inference for nonparametric logistic regression. Stat. Med..

[25] Fatima U., Hina S., Wasif M. (2023). A novel global clustering coefficient-dependent degree centrality (GCCDC) Metric for large network analysis using real-world datasets. J. Comput. Sci..

[26] Palanivinayagam A., Damaševičius R. (2023). Effective handling of missing values in datasets for classification using machine learning methods. Information.

[27] Chan J.Y.L., Leow S.M.H., Bea K.T., Cheng W.K., Phoong S.W., Hong Z.W., Chen Y.L. (2022). Mitigating the multicollinearity problem and its machine learning approach: a review. Mathematics.

